# Bioinspired Design of Sericin/Chitosan/Ag@MOF/GO Hydrogels for Efficiently Combating Resistant Bacteria, Rapid Hemostasis, and Wound Healing

**DOI:** 10.3390/polym13162812

**Published:** 2021-08-21

**Authors:** Meng Zhang, Dong Wang, Nana Ji, Shaoxiang Lee, Guohui Wang, Yuqi Zheng, Xin Zhang, Lin Yang, Zhiwei Qin, Yang Yang

**Affiliations:** 1 College of Environment and Safety Engineering, Qingdao University of Science and Technology, Qingdao 266042, China; A903962566@163.com (M.Z.); wd_charrel@163.com (D.W.); Nannaji1256@163.com (N.J.); 17864298538@163.com (G.W.); marcella_Zheng@163.com (Y.Z.); 13176388366@163.com (X.Z.); 2 Shandong Engineering Research Center for Marine Environment Corrosion and Safety Protection, Qingdao University of Science and Technology, Qingdao 266042, China; 3 Shandong Engineering Technology Research Center for Advanced Coating, Qingdao University of Science and Technology, Qingdao 266042, China; 4 Sinochem Chemical Science and Technology Research Institute Co., Ltd., Beijing 100089, China; yanglin@chemchina.com (L.Y.); qinzhiwei01@sciences.chemchina.com (Z.Q.); 5 National Marine Data and Information Service, Tianjin 300171, China; yangyangouc@163.com

**Keywords:** biomimetic synthesis, hemostasis, antibacterial activity, wound healing

## Abstract

Due to the spread of drug-resistant bacteria in hospitals, the development of antibacterial dressings has become a strategy to control wound infections caused by bacteria. Here, we reported a green strategy for in situ biomimetic syntheses of silver nanoparticles@organic frameworks/graphene oxide (Ag@MOF–GO) in sericin/chitosan/polyvinyl alcohol hydrogel. Ag@MOF–GO was synthesized in situ from the redox properties of tyrosine residues in silk sericin without additional chemicals, similar to a biomineralization process. The sericin/chitosan/Ag@MOF–GO dressing possessed a high porosity, good water retention, and a swelling ratio. The hemolysis rate of the composite was 3.9% and the cell viability rate was 131.2%, which indicated the hydrogel possessed good biocompatibility. The composite also showed excellent lasting antibacterial properties against drug-sensitive and drug-resistant pathogenic bacteria. The composite possessed excellent hemostatic activity. The coagulation effect of the composite may be related to its effect on the red blood cells and platelets, but it has nothing to do with the activation of coagulation factors. An in vitro cell migration assay confirmed and an in vivo evaluation of mice indicated that the composite could accelerate wound healing and re-epithelialization. In summary, the composite material is an ideal dressing for accelerating hemostasis, preventing bacterial infection, and promoting wound healing.

## 1. Introduction

Skin is the largest organ covering the entire body that can maintain internal balance and prevent external microbial invasion. Our body can repair damaged tissue on its own, which is a time-consuming process. However, multiple factors can cause serious skin injuries, leading to wound infection, uncontrolled bleeding, and serious complications [1,2,3]. Thus, the development of novel wound dressings with multifunctional properties, including hemostasis, low-hemolysis, long-term antibacterial activity, and wound healing, is vitally important [4,5,6].

Silk sericin, a glycoprotein that accounts for almost 30% of silk cocoons, is usually discarded from the textile industry [7]. The biological activity of sericin in improving cell adhesion, antibacterial activity, and biodegradation has also been explored as a serum substitute [8,9,10]. In addition, sericin-based hydrogels also have other properties that are beneficial to wound healing such as biocompatibility, water retention, and antioxidants [11]. However, the amorphous and fragile nature of sericin limits its application in the field of biomedicine [12]. Improving the mechanical properties of silk glue is therefore the key to expanding its potential applications. The amino, carboxyl, and hydroxyl functional groups in sericin enable it to be successfully blended or cross-linked with other polymers, thus improving its mechanical properties. Polyvinyl alcohol (PVA) is a polymer with excellent properties such as gas permeability, biocompatibility, and excellent film forming ability, and is often blended with natural polymers to improve their mechanical properties [13,14,15]. Chitosan is another attractive biopolymer as it promotes the generation of an extracellular matrix and the activation of macrophages to accelerate wound healing [16,17,18,19,20,21]. Blending silk gum with PVA could improve the mechanical properties of silk gum while maintaining the swelling ability of chitosan, thus expanding the application of sericin-based composites in the biomedical field. However, the spread of antibiotic-resistant bacteria in hospitals often leads to wound infection, which reduces the application effect of sericin-based composite dressings in the care of infected wounds [22]. At present, many studies have focused on the development of more effective sericin-based composites with antibacterial activity to treat wound infections related to multi-antibiotic resistant bacteria [23,24,25].

Metal–organic frameworks (MOFs) are a new type of coordination polymer formed by the bonding of metal cations and organic ligands [26,27,28]. MOFs have been widely used in the medical field because of their application in different areas such as drug delivery [29], wound healing [30], bioimaging [31], and biosensors [32] to improve the human lifestyle. Therefore, to speed up healing and prevent microorganisms from entering the wound, MOFs are used as fillers for wound healing [33]. In recent years, Ag–Metal organic frameworks (Ag–MOF) have attracted more and more attention based on the strong antibacterial activity of silver [34]. Many studies have proven that silver is a powerful weapon against drug-resistant bacteria [35]. However, the three-dimensional structure of Ag–MOF is easily destroyed in water, which limits its application in the field of medical dressing hydrogels. The preparation of silver nanoparticles@organic frameworks (Ag@MOF) through the organic ligand adsorption of silver nanoparticles has become an effective method to solve the problem. In addition, organic ligands of Ag@MOF can enhance their biocompatibility and can help Ag^+^ penetrate the bacterial cell membrane [36,37]. The three-dimensional structure of the organic ligand can be used as a metal ion library to realize the slow release of Ag^+^, which can keep the wound sterile for a long time [38]. However, some studies have also shown that high concentrations of Ag@MOF have a certain cytotoxicity and may cause DNA damage [36].

Graphite oxide (GO) is an oxidized form of the graphene sheet, and oxygenated functional groups are attached to the substrate planes and edges of GO sheets [39,40,41,42]. As a result, this makes GO more hydrophilic with a larger surface area [43]. Its biocompatibility is mainly affected by its high surface area and hydrophilicity, which makes GO more widely used in the field of biomedicine [44,45]. GO has good antibacterial activity [36]. In order to use Ag@MOF safely, supporting Ag@MOF with Go is an effective way to reduce the amount of Ag@MOF without affecting its functions. Moreover, the formation of Ag@MOF–GO composites promoted synergistic effects which can improve their antibacterial activity compared to their original components.

In previous reports, AgNPs–GO was immobilized in polyvinyl alcohol or chitosan by chemical or physical adsorption after synthesis through a complex process [23]. The green synthesis of AgNPs–GO has received more and more attention due to the increasing awareness of environmental protection. Biomineralization is the combination of nanotechnology and biology, which uses a biomacromolecule to synthesize a biocomposite [9]. This provided inspiration for researchers to design advanced biocomposites and nanocomposites. In the past few decades, natural polymers have been usually used as a template to form inorganic–organic hybrid composite materials [46]. Biological materials inspired by proteins such as sericin have attracted more and more interest due to their fascinating properties and practical applications [9].

**Figure 1:**
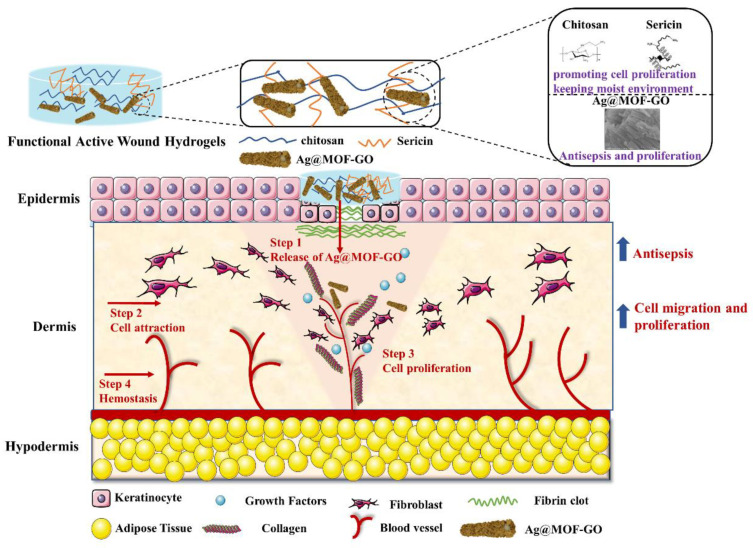
The design of chitosan/silk sericin (CS/SS) hydrogels incorporated with silver nanoparticles@organic frameworks/graphene oxide (Ag@MOF–GO).

Our work aimed to prepare CS/SS/Ag@MOF–GO hydrogels with hemostasis, antibacterial, cell adhesion, low toxicity, and wound healing as seen in Figure 1. Ag@MOF–GO impregnated into sericin/chitosan (SS/CS) hydrogels were successfully synthesized through a green strategy. The microstructure, water-solubility, swelling degree, and water retention of hydrogels were characterized. Furthermore, the hemostatic activity, antibacterial activity, biocompatibility, cell adhesion, cell migration, and animal experiments were evaluated systematically.

## 2. Materials and Methods

### 2.1. Materials

Chitosan (with a degree of deacetylation 96.56%), polyvinyl alcohol (PVA: with an average degree of polymerization of 1788), and 1,3,5-benzenetricarboxylic acid were provided by Aladdin reagent Shanghai Co., Ltd. (Shanghai, China). Sericin, AgNO_3_, NaOH, and glycerol were procured from Sinopharm Chemical Reagent Co., Ltd. Beijing, China. Graphite flakes were obtained from Sigma Aldrich (St. Louis, MO, USA). The reagents used were all of analytical grade.

### 2.2. Synthesis of Ag@MOF

First, 1.025 g 1,3,5-benzenetricarboxylic acid (BTC) was dispersed in 75 mL of deionized water. A NaOH solution (1 M) was added dropwise to the suspension until the pH value reached 7. In this solution, 12.5 mL AgNPs solution (0.05 wt%) was also added dropwise and stirring was continued for 24 h. The solution was washed 3 times alternately with distilled water and ethanol, and dried.

### 2.3. Preparation of GO Sheets

GO was synthesized by the Hummers method [47]. Graphite flakes (2 g) were dissolved in 46 mL H_2_SO_4_ and stirred to form a black solution. A total of 1 g sodium nitrate was added to the solution in the ice bath and stirred for 3 h, then 6 g of potassium permanganate (KMnO_4_) was added as the oxidant to keep the temperature below 20 °C for 30 min. A total of 92 mL of deionized water was added to the solution, stirred for 15 min and then 80 mL of 3% H_2_O_2_ (the solution is close to brown at this time) was added. The solution was centrifuged at 7200 rpm for 30 min, and the supernatant was decanted. The resulting precipitate was centrifugally cycled in deionized water until a neutral pH value (7.0) was obtained. The sample was freeze-dried at −80 °C for 24 h.

### 2.4. Synthesis of Ag@MOF–GO

Ag@MOF–GO nanocomposites were synthesized by the deposition–precipitation method. First, 1.025 g 1,3,5-benzenetricarboxylic acid (BTC) was dispersed in 75 mL of deionized water, then 1 M of NaOH solution was added dropwise to the suspension, and 75 mL distilled water was added until the pH value reached 7. A total of 12.5 mL of AgNPs solution (0.05 wt%) and 15 mL of graphene oxide solution (0.1 wt%) was added to the suspension and stirred for 24 h. The solution was washed with alcohol and deionized water 3 times, and then dried at 60 °C for 24 h to obtain the Ag@MOF–GO composite material.

### 2.5. Preparation of CS/SS/Ag@MOF and CS/SS/GO Hydrogels

Briefly, the silkworm cocoon pieces (2 g) were boiled at 120 °C for 60 min at a high pressure, and the sericin was extracted into water (60 mL). Then, the sericin solution was lyophilized into powder after filtration to remove sericin fibers. A total of 2 g of chitosan was dissolved in 100 mL of 1.5% (v/v) acetic acid solution and stirred at room temperature for 1 h to prepare the chitosan solution. A total of 100 mL of SS solution (2% *w/v* in water) and 200 mL of PAV solution (15% *w/v* in water) was prepared, while the solution was stirred and mixed to obtain a CS/SS solution. A total of 0.5% Ag@MOF and 0.5% GO was added to the solution and stirred evenly,. The solution was frozen at −25 °C for 24 h and thawed at 25 °C for 8 h for three cycles before being washed with deionized water.

### 2.6. Preparation of CS/SS/Ag@MO–GO Nanocomposite Hydrogels

The CS/SS/Ag@MOF–GO nanocomposite was synthesized by the freeze–thaw cycle method. A total of 1.025 g of 1,3,5-benzenetricarboxylic acid (BTC) was dispersed in 75 mL of deionized water, 1 M of NaOH solution was dropped into the suspension, and 75 mL of distilled water was added until the pH reached 7. Then, 12.5 mL of silver nitrate solution (0.05 wt%), 15 mL of graphene oxide solution (0.1 wt%), and a certain amount of CS/SS solution were added to the suspension and stirred for 24 h. The solution was frozen at −25 °C for 24 h and thawed at 25 °C for 8 h for three cycles before being washed with deionized water to obtain the hydrogel.

### 2.7. Characterization

The samples Ag@MOF, GO, and Ag@MOF–GO as well as their composite hydrogels were recorded on an IRAffinity-1 spectrometer (Shimadzu, Kyoto, Japan) in the range 4000–400 cm^−1^ with a scanning rate of 2 cm^−1^. The morphology of Ag@MOF, GO, and Ag@MOF–GO as well as their composite hydrogels were obtained by a JSM-6700F scanning electron microscope (SEM) (JEOL, Tokyo, Japan). Transmission Electron Microscopy (TEM) images were investigated by a Philips CM 120 transmission electron microscope. X-ray diffraction spectra were obtained by an X-Ray diffractometer (ULTIMALV, Japan Science Corporation, Tokyo, Japan). The scanning diffraction angle was between 5 and 50°, and the current was 20 mA. UV spectroscopy was investigated by a Shimadzu UV-2550 spectrometer in the scanning mode of 200–800 nm.

### 2.8. Thermogravimetric Analysis

The thermal property of the samples was conducted using the SDT-Q600 thermal analyzer (USA). The samples were heated in a nitrogen atmosphere at 50–700 °C.

### 2.9. Water Solubility (WS) of the Hydrogels

The hydrogel was cut into 1 cm × 1 cm and dried at 110 °C to obtain the initial dry mass (W_1_). Subsequently, the hydrogel was completely immersed in distilled water for 24 h and then dried at 110 °C to obtain the final mass (W_2_). The WS was calculated by the Equation (1):

(1) $$WS=\frac{W_{1}-W_{2}}{W_{1}}\times 100$$

### 2.10. Water Vapor Permeability

The water vapor permeability (WVP) was measured by gravimetrical methodology [48]. The hydrogel was sealed in a cup containing anhydrous calcium chloride and then placed in a desiccator containing a saturated solution of deionized water to dry for 30 h. The weight of the cup was gradually increased until it reached a steady state. The WVP was calculated as follows:

(2) $$WVP=\frac{\Delta W\times d}{S\times t\times \Delta P}$$

where ΔW (g) is the weight gain of the World Cup; d (m) is the thickness of the hydrogel; S (m^2^) is the exposed hydrogel area; t (h) is the time; and ΔP (KPa) is the actual difference between the two samples.

### 2.11. Water Retention Studies

The hydrogel was soaked in distilled water at 37 °C, and the equilibrium swelled hydrogel was placed in an oven at 37 °C [49]. The hydrogel was weighed at predetermined intervals. The water retention (WR) for the hydrogel was calculated as follows:

(3) $$WR(\%)=\frac{W_{t}}{W_{e}}\times 100$$

where W_t_ is the weight of the hydrogel at time t and W_e_ is the swelling weight at equilibrium.

### 2.12. In Vitro Antibacterial Study and Ag Ion Release Measurement

The inhibition zone method was used to assess the antibacterial activity of the hydrogel with the strains of drug-sensitive *E. coli*, *S. aureus*, and drug-resistant *DRE* and *MRSA* [50]. First, 100 µL of 10^8^ CFU/mL bacterial suspension was spread on the LB agar plate, and then the sterile hydrogel with a diameter of 6 mm (sterilized by UV irradiation) was placed on the agar surface. The plate was placed in an incubator at 37 °C for 48 h, and then the diameter of the inhibition zone was measured.

A single horizontal diffusion cell was used for the skin penetration test in vitro, with an effective diffusion area of 1.13 cm^2^. The CS/SS/Ag@MOF and CS/SS/Ag@MOF–GO films (1.13 cm^2^) were applied to the stratum corneum of the skin. A phosphate buffer brine (PBS, 3.0 mL, pH 7.4) acted as the receiving body fluid. The sampling time was 2, 4, 6, 8, 10, 12, 24 h. The receptor culture medium was extracted at 2.0 mL, and the same volume of fresh receptor medium was added to keep the receptor volume unchanged. The concentration of the receiver solution was determined by HPLC.

### 2.13. Antibacterial Effects of the Extract Liquid of Samples

The liquid CS/SS/Ag@MOF–GO and CS/SS/Ag@MOF samples were extracted and a ratio of 1.25 cm^2^/mL was prepared according to the ISO 108993.12–2012, in which CS/SS/Ag@MOF-GO and CS/SS/Ag@MOF specimens were immersed in 0.9% NaCl solution at 37 °C for 24 h. A total of 10 μL of the bacterial suspension and 90 μL of the extract liquid was dropped on the petri dish, and cultured at 37 °C for 3 h, 6 h, 18 h, and 24 h. Then, the colony forming units on petri dish were counted.

### 2.14. Morphological Characterization of Bacteria

*E. coli*, *DRE*, *S. aureus*, and *MRSA* cells were incubated with CS/SS/Ag@MO–GO dispersion (65 µg/mL) in isotonic saline solution at 37 °C and were shaken at 200 rpm for 3 h [51]. Then the bacteria cells were washed 3 times with PBS and fixed with 2.5% glutaraldehyde solution for 3 h. The samples were dehydrated with 30/40/60/80/90/100% ethanol gradient solution and freeze-dried. Finally, the surface morphology of the samples was observed by the scanning electron microscope.

### 2.15. In Vitro Cytotoxicity

The L929 cells were grown in Dulbecco’s modified Eagle medium containing 10% heat-inactivated fetal bovine serum with 2 mM L-glutamine, 4.5 g L^−1^ glucose, and 1% penicillin/streptomycin solution. The cells were kept under aseptic conditions at 37 °C and 5% CO_2_. The media were refreshed every 2 days until the cells reached confluence. The disc shaped sponges (CS/SS, CS/SS/Ag@MOF, CS/SS/GO, and CS/SS/Ag@MOF–GO) were sterilized in 75% ethanol for 30 min and irradiated in ultraviolet (UV) radiation for another 30 min. The discs were placed onto a 24-well plate after rinsing with sterilized PBS (10 mM, pH 7.4) thrice and seeded with 1 mL cell suspension at 2 × 10^4^ cells mL^−1^ concentration in each well. After incubating for 24 h, 100 μL MTT solutions were added for 4 h, then 400 μL of DMSO was added to each well, and the resultant dissolvable solution was transferred into a 96-well plate. The absorbance at 490 nm was recorded using a microplate reader. The control group containing cells was cultured without samples. The cell viability of the samples was calculated as:

Cell viability = OD_490_(sample)/OD_490_(control) × 100%, where the optical density (OD) values of the samples and the control are coded as OD_490_(sample) and OD_490_(control).

### 2.16. Hemostatic Properties of Hydrogels

#### 2.16.1. Blood Clotting Kinetics and Clotting Blood Time (CBT)

In brief, 0.7 mL of anticoagulant rabbit blood from the volunteer was collected in EDTA-coated Falcon tubes. Composite dressings (5 mm × 5 mm) were placed in a polypropylene tube. Blood was slowly distributed on to the composite dressing and coagulation was initiated by adding 0.5 mL of 0.2 M calcium chloride to the blood. Further, tubes containing blood were incubated at 37 °C for 5 min, 10 min, and 15 min, and 20 mL of deionized water was slowly added through the walls of the tubes without disturbing the clotted blood. The red blood cells (RBCs) that were not entrapped in the clot were hemolyzed with water and the absorbance of the resultant RBCs containing solution was measured at 540 nm. A similar treatment was followed for a controlled sample (blank) without the composite.

Samples of 20 mg were put into the test tube, preheated at 37 °C for 5 min, and 1 mL of anticoagulant rabbit blood was added for further incubation at 37 °C for 3 min. Then 500 µL of CaCl_2_ solution (25 mmol/L) was added. The time from when the CaCl_2_ was added to when the blood clot was designated as CBT was measured. The unsampled blank group was used as the control group and the same method was used. Three measurements were taken for each group.

#### 2.16.2. Blood Plasma Clotting Analysis

Platelet-poor plasma (PPP) was obtained from anticoagulated whole blood centrifuged at 3000 rpm for 15 min. A 100 mg sample was mixed with 1 mL PPP and incubated at 37 °C with constant shaking at a constant speed for 20 min so that the sample and plasma were evenly mixed and could be used as the plasma to be tested. A total of 0.1 mL of the plasma to be tested was placed in the test tube, and then 0.1 mL of the APTT test solution was added and placed in the instrument for clotting time determination and incubated for 3 min at 37 °C. Then 0.1 mL of the 0.025 mol/L CaCl_2_ solution was added and put into the instrument to measure the coagulation time. Similarly, the corresponding kits were used to detect the prothrombin time (PT) and thrombin time (TT) [52]. Each group was measured three times.

### 2.17. Adhesion of Erythrocyte and Platelets

Samples of 100 mg were incubated in 37 °C anticoagulated whole blood for 30 min [53]. Then the samples were washed 3 times with a phosphate-buffered saline (PBS) and fixed with 2.5% glutaraldehyde solution for 3 h. After washing with PBS, it was dehydrated with 30/40/60/80/90/100% ethanol gradient solution and freeze-dried. The anticoagulated blood was centrifuged at 1000 rpm/min for 10 min, and platelet-rich plasma was obtained from the upper layer. Similarly, the adhesion of the platelets to the samples was proved by the same method. The surface of the hydrogel was observed on the scanning electron microscope.

### 2.18. Hemolysis Assay

The diluted whole blood solution was prepared by diluting 4 mL of fresh anticoagulant whole blood with 5 mL of 0.9 wt% NaCl solution [54]. The sample was cut into small pieces of 1 cm × 1 cm and rinsed with distilled water and 0.9 wt% NaCl solution. It was put into a test tube, and 10 mL of 0.9 wt% NaCl solution was added. The tube was heated at 37 °C for 30 min, then 0.2 mL diluted whole blood was added and heated at 37 °C for 1 h. The solution was centrifuged at 1000 rpm for 10 min, and then the absorbance of the supernatant at 545 nm was measured with an ultraviolet spectrophotometer. The hemolysis was calculated according to the formula:

(4) $$HR=\frac{OD_{sam}-OD_{\mathrm{neg}}}{OD_{\mathrm{pos}}-OD_{\mathrm{neg}}}$$

where OD_sam_, OD_neg,_ and OD_pos_ are the adsorptions, negative control, and positive control of samples, respectively.

### 2.19. Cell Migration Assay

L929 fibroblasts were seeded in DMEM containing 1% penicillin-streptomycin and 10% fetal bovine serum (FBS) [39]. The cells (1 × 10^4^) were seeded in a 24-well plate and incubated at 37 °C in a humidified CO_2_. They were scratched with a 10 mL pipette and rinsed with PBS to remove dead cells. The cells were treated with Ag@MOF–GO, and the other group served as a control. The cells were lightly washed again with PBS, fixed with 4% of paraformaldehyde for 25 min, and then the cells were labeled to obtain an optical image.

### 2.20. In Vivo Animal Experiment

Sixteen eight-week-old male BALB/c mice with a bodyweight of about 20 g were divided into 4 groups. The mice were anesthetized with ether on the day of injury, and a full-thickness circular wound (about 10 mm in diameter) was made on the back of the mice. Control-1 (medical gauze), CS/SS, control-2(medical gauze), and CS/SS/Ag@MOF-GO were used to cover the wound in the 4 groups. The dressings were changed daily. The wound area was measured and photographed when the dressing was changed. The image J was used to measure the wound area. The picture file was dragged to the software area and opened quickly. The picture intended to be processed was selected, and the distance between the ruler and the wound adjusted, then the area with the appropriate area was selected, and the pictures were cropped outside the selected area. The image brightness and contrast were adjusted and the color threshold was adjusted, and the wound area was selected by changing the threshold. The “Magic Wand” was clicked on in the menu bar and the selected area was clicked on to automatically generate ROI. The wound area with our own sketch was selected and stored in the ROI manager. The ROI area was calculated and the data was saved. The healing rate (HR) was calculated as follows:

(5) $$HR=\frac{S_{0}-S}{S_{0}}\times 100\%$$

In the formula, *S*_0_ and *S* are, respectively, the wound area on the day when the wound surface was formed and the wound area on the day when the dressing was added.

A total of 14 days after the injury, a sample of skin tissue was taken, fixed with 10% formalin, and a histological examination was performed. The samples were stained with hematoxylin–eosin (H&E) and observed under a Leica DMI3000B light microscope (Sigma Aldrich, St. Louis, MO, USA).

### 2.21. Statistical Analysis

All experiments were carried out 3 times (*n* = 3), and the data are expressed as mean ± standard deviation. The results were analyzed with a one-way ANOVA by SPSS (version 22.0, SPSS Inc., Chicago, IL, USA).

## 3. Results and Discussion

### 3.1. Characterization of Ag@MOF–GO

**Figure 2:**
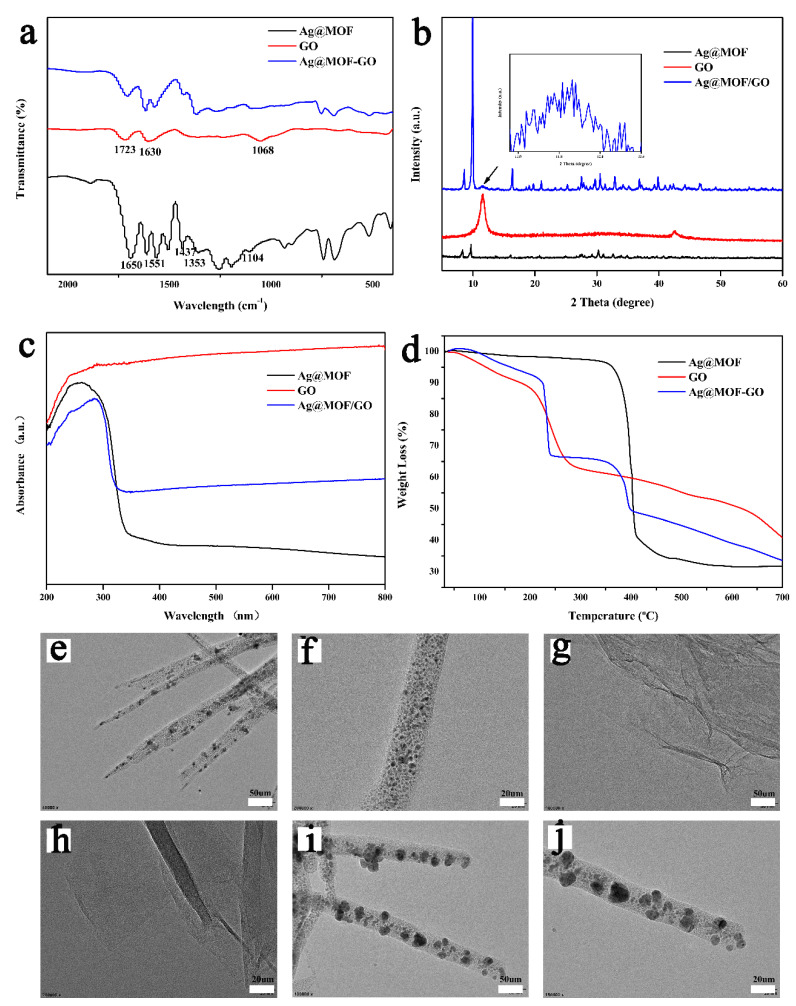
(**a**) FTIR spectrum, (**b**) XRD pattern, (**c**) UV–vis spectrum, (**d**) TGA curves of Ag@MOF, GO, and Ag@MOF–GO; TEM images of (**e**,**f**) Ag@MOF, (**g**,**h**) GO, and (**i**,**j**) Ag@MOF–GO.

The FTIR spectrum of the synthesized Ag@MOF, GO, and Ag@MOF–GO are shown in Figure 2a. In the spectrum of the initial Ag@MOF, the bands between 1250 cm^−1^ and 1750 cm^−1^ corresponded to the carboxylic acid ligands of BTC coordinated with metal sites of the framework [36]. The symmetric and asymmetric stretching vibration modes of the carboxylate groups in BTC were observed at 1353/1437 cm^−1^ and 1551/1650 cm^−1^, respectively. The band at 1104 cm^−1^ is assigned to the C-O stretching vibration of the C-OH group. In the case of GO, the band at 1723 cm^−1^ is assigned to C=O stretching vibration on the carboxyl group while the absorption band at 1630 cm^−1^ may be the bending vibration of C-OH [39]. The band at 1068 cm^−1^ is attributed to the stretching vibration modes of C-O-C. Ag@MOF–GO has similar characteristics to Ag@MOF. Both Ag@MOF and GO contain carboxyl groups. However, the changes in the environment of the carboxylic acid ligands indicate that the interaction of ligands with GO led to a distortion of the MOF framework. The decreased intensity in the bands between 1250 and 1750 cm^−1^ may be related to the coordination change of the carboxylic acid ligand, which may be due to the functional group of GO participating in MOF as linkers. The slight shift of the band corresponding to the BTC carboxylic acid ligand (between 1250 and 1750 cm^−1^) to the right also proves the change in the coordination of the carboxylic acid ligand of MOF. Due to the complete absence of C-O-C corresponding to the band at 1068 cm^−1^, it supports the formation of Ag@MOF–GO composites instead of physical mixtures of components. Considering that this group and the carboxyl group can easily coordinate with the Ag^+^ center and occupy the surrounding coordination sites, they are likely to participate in the formation of the composite.

Figure 2b shows the X-ray diffraction (XRD) patterns of the synthesized Ag@MOF, GO, and Ag@MOF–GO. The large d-spacing of the diffraction peaks of GO at the 11.12 (001) plane indicates that oxygen-containing functional groups were attached to the structure during the oxidation process [55]. A lower intensity peak appeared at 26.49 which indicates the presence of a small amount of reduced graphene oxide. The XRD patterns of the Ag@MOF–GO composites are very similar to those of Ag@MOF and show characteristic peaks, which are related to the crystal structure of the MOF phase. Furthermore, the crystal structure of Ag@MOF–GO is disturbed to a certain extent, which may be related to the coordination of the C-O-C group to the Ag^+^ center. The result is consistent with FTIR analysis.

The UV–visible spectrum of the synthesized samples is shown in Figure 2c. In the case of GO, the absorption band at 242 nm was similar to the π-πtransition of the aromatic C-C bond, while the band at 292 nm was related to the n-π * transition of the C=O bond [39]. For Ag@MOF, the absorption band at 256 nm corresponds to the π-π * transition of the aromatic C-C bond. The slight shift of the absorption bands of GO to the right is due to the faster electron transfer speed and the increase in the transition energy between the molecules of the Ag@MOF–GO nanocomposite.

The TEM images of the synthesized samples are presented in Figure 2. Ag@MOF is composed of many long rod-shaped nanoparticles with diameters ranging from 100 to 400 nanoparticles [36]. The silver component in Ag@MOF (Figure 2e,f) is well dispersed, mainly with a size of about 7 nm in diameter. Synthetic graphene oxide has a curved and folded structure, and there are some cracks (Figure 2g,h) [43]. The addition of GO (Ag@MOF–GO) resulted in the formation of longer, regular rods with a diameter almost twice that of the MOF itself (Figure 2i,j). In addition, compared with Ag@MOF, the size of the Ag component in Ag@MOF–GO is larger. Interestingly, the surface of the composite rod (Ag@MOF–GO) has a lot of perforations. This structure may have a positive impact on the properties of the nanocomposite and further promote the release of silver in the Ag@MOF–GO when it comes into contact with bacteria.

### 3.2. FTIR of Composite Dresssings

**Figure 3:**
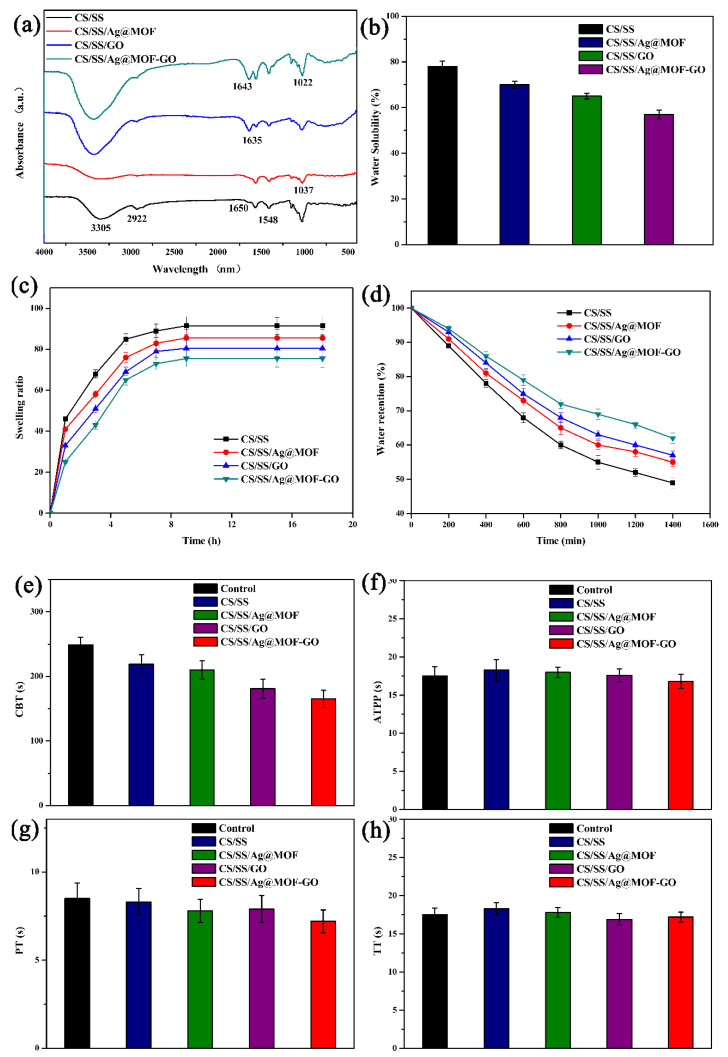
(**a**) FTIR spectrum, (**b**) water solubility, (**c**) swelling degree, (**d**) water retention, (**e**) CBT, (**f**) APTT, (**g**) PT, and (**h**) TT of CS/SS, CS/SS/Ag@MOF, CS/SS/GO and CS/SS/Ag@MOF–GO.

The infrared spectrum of the CS/SS hydrogels showed the characteristic bands of sericin and chitosan, which may be due to the presence of the intermolecular and intramolecular hydrogen bonding networks between chitosan and sericin. Figure 3a shows that the absorption band at 3305 cm^−1^ corresponds to the stretching vibration of -NH and -OH while the absorption band at 2922 cm^−1^ is assigned to the asymmetric stretching vibration of the CH bond [56,57]. The band at 1650 cm^−1^ may be related to the C=O stretching vibration of amide I while the absorption band at 1548 cm^−1^ and 1408 cm^−1^ may be assigned to the -OH and -NH bending vibration of amide II, respectively [50]. After the addition of Ag@MOF, the shift of the band at 1037 cm^−1^ to the left may be due to the Ag-O stretching vibration mode. With the addition of GO, the composite also showed a new absorption band around 1635 cm^−1^, indicating the bending vibration of C-OH. Similarly, for the CS/SS/Ag@MOF–GO composite, the sharp bands near 1643 cm^−1^ and 1022 cm^−1^ are considered to be Ag@MOF–GO located in the CS/SS.

### 3.3. Water Solubility, Swelling Degree, and Water Retention

Figure 3b–d shows the results of the moisture-related properties. CS/SS, CS/SS/Ag@MOF, CS/SS/GO, and CS/SS/Ag@MOF–GO showed water solubility and a swelling degree in descending order while they exhibited water retention in ascending order. This may be due to the rigid hydrophobic structure of Ag@MOF and the layered structure of GO [39]. The lower swelling and water solubility of the wound dressing can prevent the dressing from dissolving or deforming and causing negative effects on the wound [58]. The higher water retention provides a moist environment for the wound and promotes wound healing [49].

### 3.4. Hemostatic Properties of Dressings

**Figure 4:**
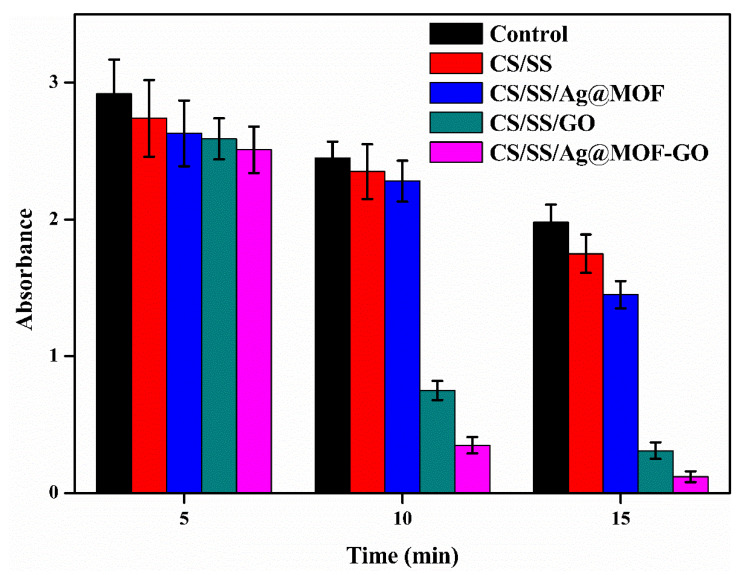
Blood clotting kinetics of the composite dressing compared to the control.

The hemostatic activity of the composite dressing was evaluated by determining the absorbance value of RBCs at 540 nm in un-coagulated blood. As seen in Figure 4, CS/SS, CS/SS/Ag@MOF, CS/SS/GO, and CS/SS/Ag@MOF–GO showed absorbance values in descending order. This indicated that the CS/SS/Ag@MOF–GO dressing had a higher adsorption efficiency as compared to the control blood without the dressing. Thus, the CS/SS/Ag@MOF–GO composite dressing had a faster blood clotting ability, compared to the blank (without dressing), as shown in Figure 4. CBT is an effective method to determine the blood coagulation performance of materials. The hemostatic performance of the samples was intuitively evaluated through the CBT test. Figure 3e shows that the time of clot formation for the control group (blood only) was nearly 249 s, and about 219, 210, 181 s for CS/SS, CS/SS/Ag@MOF, CS/SS/GO, respectively, while that for CS/SS/Ag@MOF–GO was no more than 160 s. The results indicate that CS/SS/Ag@MOF–GO had significant advantages over the other samples in promoting blood coagulation.

The plasma coagulation cascade consists of internal, external, and common pathways, all of which are gathered at a common point, where factor X is activated to XA, which in turn activates prothrombin to thrombus [59]. APTT is a relatively common screening experiment for the endogenous coagulation system, which is used to determine the endogenous pathway coagulation factors [52]. PT is generally considered to be an effective method for evaluating the exogenous coagulation system. TT is used for the determination of thrombin in plasma in vitro to check the ability of fibrinogen to convert to fibrin (common pathway). Figure 3f–h shows the test results of APTT, PT, and TT of each material after contact with the rabbit plasma. It can be seen that for all the experimental groups, APTT was between 16.8 s and 18.3 s, PT was between 7.2 s and 8.5 s, and TT was between 16.2 s and 18.4 s, which showed that there was no significant difference between the samples. Compared with the measurement time, there was no significant difference. This result shows that the hemostatic effect of CS/SS/Ag@MOF–GO was not achieved through traditional internal, external, and common pathways, which is in line with the results of other polymers containing GO. The process of blood clotting mainly depends on the effect of the coagulation factors as well as the quality and quantity of effective erythrocytes and platelets. Since CS/SS/Ag@MOF–GO did not show the effect of promoting coagulation factors, this may be due to thrombosis caused by the effect of CS/SS/Ag@MOF–GO on erythrocytes and platelets.

### 3.5. Adhesion of Erythrocyte and Platelets

**Figure 5:**
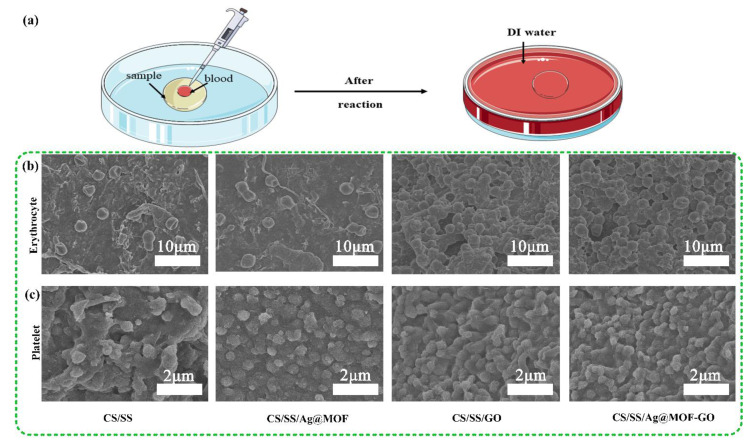
(**a**) Schematic illustration of the in vitro clotting test; SEM images of (**b**) erythrocyte and (**c**) platelet adhesion to the samples.

By observing the number and morphology of erythrocytes and platelets that adhered to the surface of the material, the hemostatic mechanism of the sample was further studied. The results (Figure 5b,c) show that most of the platelets that adhered to each sample were deformed, indicating that they had an outstanding efficiency in activating platelets [53]. The hydrogel did not affect the normal morphology and activity of erythrocytes, indicating that it has a good biocompatibility to erythrocytes. In addition, the number of erythrocytes and platelets that adhered to the surface of CS/SS, CS/SS/Ag@MOF, CS/SS/GO, CS/SS/Ag@MOF–GO was in increasing order, which explains the difference in CBT for these groups. Compared with the other samples, Ag@MOF–GO in CS/SS/Ag@MO–-GO has a compact structure and larger specific surface area, allowing more erythrocytes and platelets to adhere and aggregate to accelerate blood coagulation, which is consistent with the results of SEM.

### 3.6. Antibacterial Activities and Ag Ion Release of the Dressings

**Figure 6:**
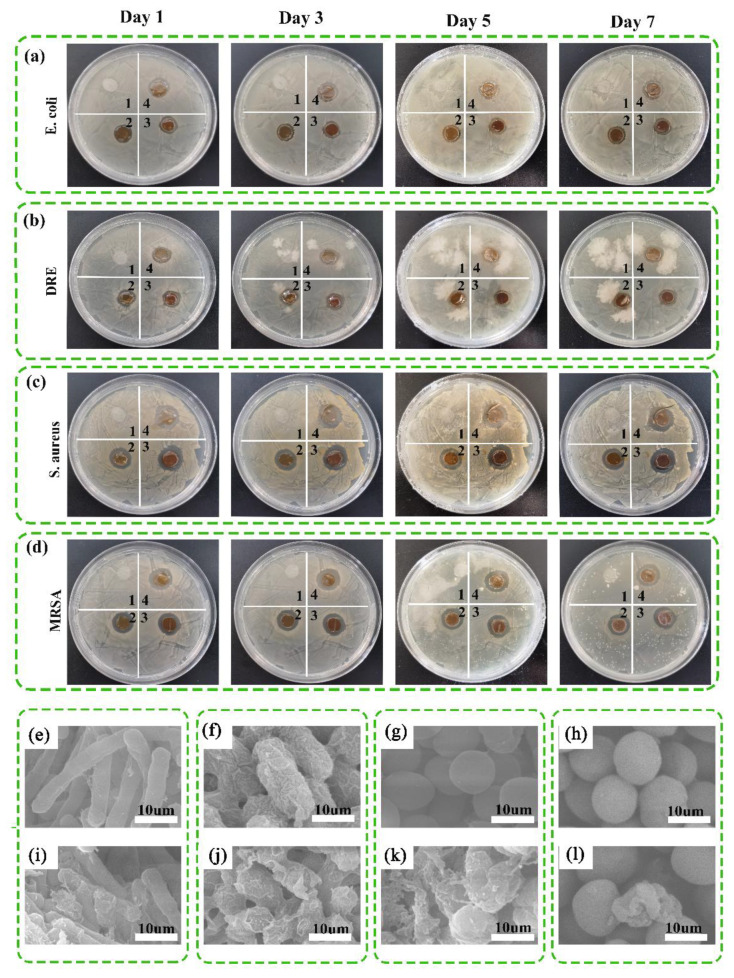
Pictures of the inhibition zones for bacterial from the sterilizing effectiveness test for (**a**) *E. coli*, (**b**) *DRE*, (**c**) *S. aureus*, and (**d**) *MRSA*; (1) CS/SS, (2) CS/SS/Ag@MOF, (3) CS/SS/GO, and (4) CS/SS/Ag@MOF–GO. SEM images of intact and damaged *E. coli* (**e**,**i**), *DRE* (**f**,**j**), *S. aureus* (**g**,**k**), and *MRSA* (**h**,**l**).

In order to observe the sustained antibacterial activity of CS/SS/Ag@MOF–GO against drug-sensitive and drug-resistant pathogenic bacteria, the inhibition zone method was performed on the strains of drug-sensitive *S. aureus*, *E. coli*, and drug-resistant *MRSA*, *DREC* [60]. Figure 5 shows that CS/SS had no obvious antibacterial activity against all strains, while the inhibition zone formed by CS/SS/GO was smaller, indicating that it had a limited toxicity to bacteria. CS/SS, CS/SS/GO, CS/SS/Ag@MOF, and CS/SS/Ag@MOF–GO showed antimicrobial activity in ascending order. The antibacterial activity of CS/SS/Ag@MOF–GO was significantly increased, possibly due to the synergistic effect of the composite (Ag@MOF–GO) [36]. Ag@MOF–GO continuously released Ag^+^ to the surrounding environment, and it inactivated the bacteria in their respective regions, resulting in no significant change in the size of the inhibition zone for 7 days (Figure 6a–d).

Through SEM analysis, the nature and extent of the damage to the bacterial cell by CS/SS/Ag@MOF–GO can be recognized from the changes in the morphology of the bacteria [51]. Untreated E. coli (Figure 5e) have normal rod shapes, while after incubating *E. coli* with the sample, the cell showed severe morphological damage and shrank (Figure 5i). The remaining types of bacteria after incubation with CS/SS/Ag@MOF–GO had varying degrees of damage (Figure 6f–h,j–l).

According to our experimental results, a possible antibacterial mechanism of CS/SS/Ag@MOF–GO was further illustrated. Ag@MOF–GO in CS/SS/Ag@MOF–GO diffused to the surface of the bacteria and continuously released Ag^+^. The direct interaction between Ag^+^ and the thiol group protein may destroy the integrity of the bacterial membrane [34]. The interaction between the O-containing functional groups of CS/SS/Ag@MOF–GO and bacterial lipopolysaccharide promoted the interaction between Ag^+^ and bacteria to destroy bacterial cells. In addition, these O-containing functional groups can bond with cell cations (Ca^2+^ and Mg^2+^). In addition, the generation of reactive oxygen species (ROS) leads to the fragmentation of DNA, resulting in bacterial cell death [36].

**Figure 7:**
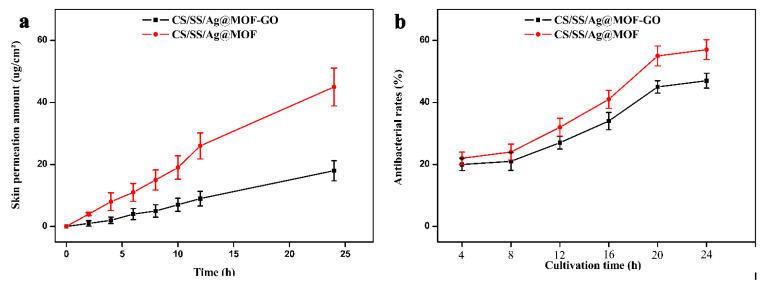
(**a**) Cumulative skin permeation profiles of Ag. (**b**) Antibacterial rates of the extracts of Ti–Ag samples.

The results shown in Figure 7a demonstrate that CS/SS/Ag@MOF possessed the maximum skin permeation effect. In addition, the skin permeation amount of CS/SS/Ag@MOF–GO and CS/SS/Ag@MOF was 18.19 and 46.23 μg/cm^2^, respectively, at 24 h. The cytotoxicity is marginal when the amount of skin penetration is small. Figure 7b shows the antibacterial effect of the extract of CS/SS/Ag@MOF and CS/SS/Ag@MOF–GO samples. It can be seen clearly that all extracts exhibited excellent antibacterial activity at all intervals at more than 20%, and CS/SS/Ag@MOF showed a higher antibacterial ability. This is due to the higher Ag^+^ release in CS/SS/Ag@MOF.

### 3.7. Biocompatibility of Dressings

**Figure 8:**
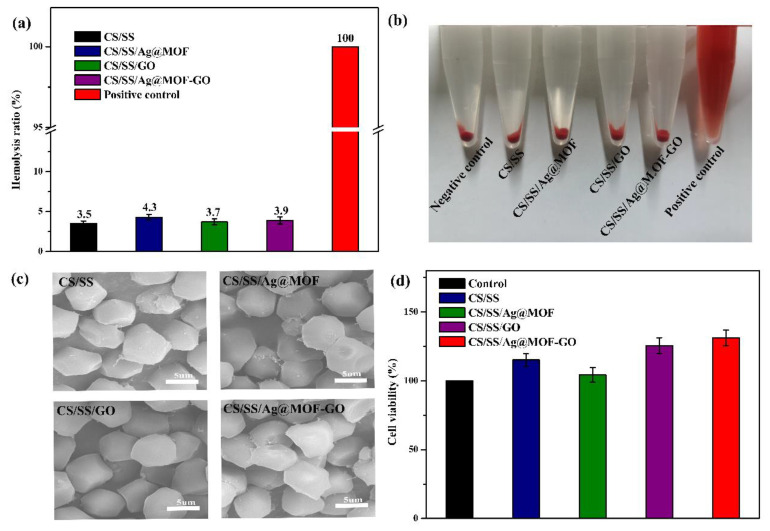
(**a**) Hemolytic percentage of the hydrogels; (**b**) Pictures from the hemolytic activity test of the hydrogels; (**c**) SEM images from the red blood cells morphology treated with the hydrogels; (**d**) Cell viability evaluation of the hydrogels.

Good biocompatibility is an important factor in wound dressings [54]. A hemolysis test was examined for an assessment of the blood compatibility of the sample. Figure 6b shows the color difference between the four sample groups, the positive control group, and the negative control group. Light yellow was observed in all four sample groups, similar to the negative control group, while bright red was observed in the positive control group. The result is shown in Figure 8a, and after adding Ag@MOF, the hemolysis rate of CS/SS was slightly increased (4.3%). Compared with the CS/SS/Ag@MOF, CS/SS/GO and CS/SS/Ag@MOF–GO showed a very low hemolysis ratio (3.7% and 3.9%), which indicated that the blood compatibility of samples was good. SEM (Figure 8c) also exhibited clearly that the structure of erythrocyte treated by samples was not damaged. Therefore, the CS/SS/Ag@MOF–GO composite dressing is considered to be a non-hemolytic material.

In order to further evaluate the cytocompatibility of these samples, the samples were tested for cytotoxicity [49]. As shown in Figure 8d, the cell viability of CS/SS, CS/SS/Ag@MOF, CS/SS/GO, CS/SS/Ag@MOF–GO was 115.1, 104.3, 125.5 and 131.2%, respectively. The CS/SS dressing obviously promoted the growth of L929 cells. Although the cell viability treated with CS/SS/Ag@MOF composite decreased due to the addition of Ag@MOF, it was still higher than the control group. The addition of GO and Ag@MOF–GO significantly enhanced the cell viability of the CS/SS composite. The above experiments confirmed that the CS/SS/Ag@MOF–GO composite has a good biocompatibility and broad application prospects as hemostatic and wound healing materials.

### 3.8. In Vitro Cell Migration Assay of Dressings

**Figure 9:**
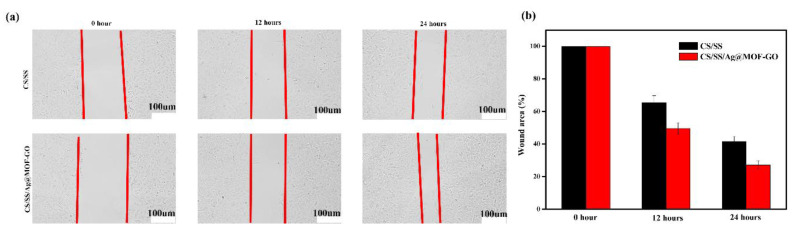
(**a**) Migration assay of L929 cells in presence of CS/SS or CS/SS/Ag@MOF-GO; (**b**) wound area (%).

The growth and migration of cells is an important feature of the tissue regeneration stage in the wound healing process [39,61]. Figure 9 shows the cell migration assay of L929 cells. It can be observed that, compared with the control group (CS/SS), CS/SS/Ag@MOF–GO exhibited an excellent cell migration ability. In addition, the prepared Ag@MOF–GO has a porous spatial structure, which is conducive to the adhesion, migration, and proliferation of cells. Figure 9b shows the quantification of the wound closure area. At the 12th and 24th hours, the wound area reduction in the presence of Ag@MOF–GO was very significant, compared with the control group. Therefore, the migration test is consistent with the higher cell migration of synthetic CS/SS/Ag@MOF–GO than the control, providing a way for potential wound healing applications.

### 3.9. In Vivo Wound Healing

**Figure 10:**
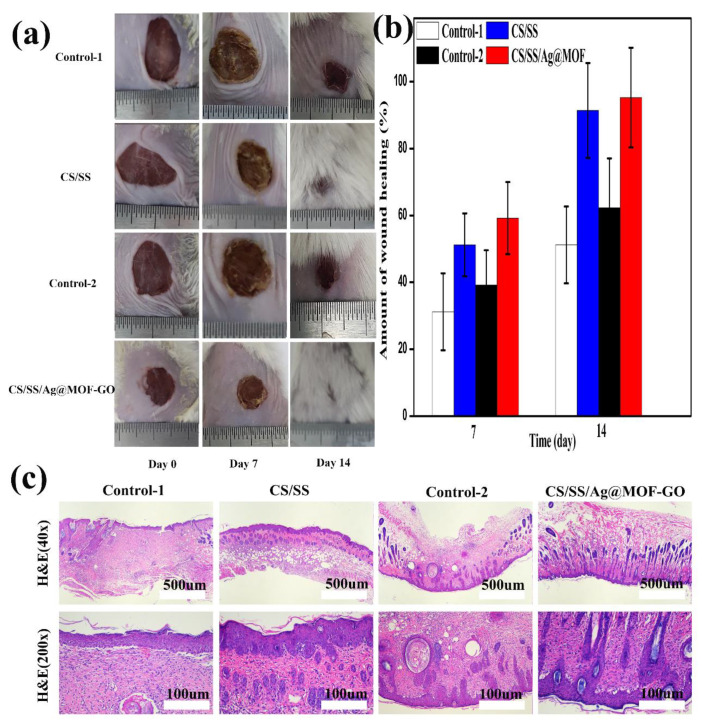
Photographs of wounds treated with the dressings, (**a**) wound treated with control-1, CS/SS, control-2, and CS/SS/Ag@MOF–GO for days, and (**b**) evaluation of the wounds area closure at 7 and 14 days; Micrographs of wound tissues stained with H&E (**c**).

Full-thickness skin wound healing was performed using CS/SS and gauze (control-1), CS/SS/Ag@MOF–GO, and commercial hemostat (control-2), and the healing rate was evaluated. Figure 10a shows that CS/SS and CS/SS/Ag@MOF–GO healed faster than control-1 and control group-2, respectively, on the 7th day after surgery. As reported in previous studies, chitosan/sericin dressings can accelerate epithelialization [10]. The CS/SS/Ag@MOF–GO and CS/SS have also been proven to have good healing effects. As shown in Figure 10b, CS/SS/Ag@MOF–GO had a significant healing rate compared to the control group-2. This good wound healing rate was attributed to the antibacterial properties of Ag@MOF-GO. On the 14th day after surgery, the wound healing rates of CS/SS and CS/SS/Ag@MOF–GO were about 91.4% and 95.3%, while those of the control group were only 51.2% and 62.3%. In general, the wound healing ability of CS/SS/Ag@MOF–GO and CS/SS was significantly better than that of gauze. These wound dressings, especially CS/SS/Ag@MOF–GO dressings, are more effective in accelerating wound healing.

### 3.10. Histological Analysis

In order to evaluate the speed and quality of the newly regenerated tissues, H&E staining was performed for histological analysis. Figure 10c shows the histological observation of the wound tissue after 14 days. The wounds treated by CS/SS and CS/SS/Ag@MOF–GO showed an intact epidermis, while the wounds treated by Control-1 and Control-2 were not completely closed. For the CS/SS and CS/SS/Ag@MOF–GO groups, the granulation tissue had been organized into fibrous connective tissue, and the epidermal density of CS/SS/Ag@MOF-GO was higher. The above results show that the CS/SS/Ag@MOF–GO dressing enhanced the ability of wound tissue repair.

## 4. Conclusions

A CS/SS/Ag@MOF–GO nanocomposite was successfully prepared through a one-step environmentally friendly method. For comparison, CS/SS/Ag@MOF and CS/SS/GO were also fabricated. The effects of the presence of GO and Ag@MOF on the microstructure, water-solubility, swelling degree, water retention hemostatic activity, antibacterial activity, biocompatibility, cell adhesion, cell migration, and animal experiment of the samples were investigated. The FTIR results showed the integrity of the structures of Ag@MOF–GO within the hydrogels were maintained well. The moisture-related properties of CS/SS were enhanced when Ag@MOF and GO were added, and it was more particularly noticeable in the presence of Ag@MOF–GO. Cytotoxicity and hemolysis tests as well as an in vitro blood clotting assay indicated that the material possessed good biocompatibility and highly effective coagulation function. However, coagulation did not play a role in the traditional model, involving exogenous, endogenous, or common pathways, which indicated that CS/SS/Ag@MOF–GO did not contribute much to the activation of coagulation. The acceleration of blood coagulation by CS/SS/Ag@MO–GO may be related to its adhesion to erythrocytes and platelets. The antibacterial test indicated that the hydrogels possessed long-lasting antibacterial activities against drug-resistant and drug-sensitive pathogenic bacteria. The in vitro cell migration assay confirmed that the CS/SS/Ag@MOF–GO dressings led to a faster cell migration compared with that of other controls. The in vivo evaluation of mice indicated that CS/SS/Ag@MOF–GO can accelerate wound healing and re-epithelialization. The above results show that the prepared CS/SS/Ag@MOF–GO composite dressing has potential application in wound care.

## Data Availability

Not applicable.

## References

[1] Khorasani M.T., Joorabloo A., Moghaddam A., Shamsi H., MansooriMoghadam Z. (2018). Incorporation of ZnO nanoparticles into heparinised polyvinyl alcohol/chitosan hydrogels for wound dressing application. Int. J. Biol. Macromol..

[2] Wang L., Yang K., Li X., Zhang X., Zhang D., Wang L.N., Lee C.S. (2021). A double-crosslinked self-healing antibacterial hydrogel with enhanced mechanical performance for wound treatment. Acta Biomater..

[3] Qian Y., Xu C., Xiong W., Jiang N., Zheng Y., He X., Ding F., Lu X., Shen J. (2021). Dual cross-linked organic-inorganic hybrid hydrogels accelerate diabetic skin wound healing. Chem. Eng. J..

[4] Jayakumar R., Prabaharan M., Sudheesh Kumar P.T., Nair S.V., Tamura H. (2011). Biomaterials based on chitin and chitosan in wound dressing applications. Biotechnol. Adv..

[5] Thi P.L., Lee Y., Tran D.L., Thi T.T.H., Kang J.I., Park K.M., Park K.D. (2020). In situ forming and reactive oxygen species-scavenging gelatin hydrogels for enhancing wound healing efficacy. Acta Biomater..

[6] Zandi N., Dolatyar B., Lotfi R., Shallageh Y., Shokrgozar M.A., Tamjid E., Annabi N., Simchi A. (2021). Biomimetic nanoengineered scaffold for enhanced full-thickness cutaneous wound healing. Acta Biomater..

[7] Cao T.T., Zhang Y.Q. (2016). Processing and characterization of silk sericin from Bombyx mori and its application in biomaterials and biomedicines. Mater. Sci. Eng. C Mater. Biol. Appl..

[8] Gilotra S., Chouhan D., Bhardwaj N., Nandi S.K., Mandal B.B. (2018). Potential of silk sericin based nanofibrous mats for wound dressing applications. Mater. Sci. Eng. C Mater. Biol. Appl..

[9] Tao G., Cai R., Wang Y., Liu L., Zuo H., Zhao P., Umar A., Mao C., Xia Q., He H. (2019). Bioinspired design of AgNPs embedded silk sericin-based sponges for efficiently combating bacteria and promoting wound healing. Mater. Des..

[10] Bakhsheshi-Rad H.R., Ismail A.F., Aziz M., Akbari M., Hadisi Z., Omidi M., Chen X. (2020). Development of the PVA/CS nanofibers containing silk protein sericin as a wound dressing: In vitro and in vivo assessment. Int. J. Biol. Macromol..

[11] Chouhan D., Mandal B.B. (2020). Silk biomaterials in wound healing and skin regeneration therapeutics: From bench to bedside. Acta Biomater..

[12] He H., Cai R., Wang Y., Tao G., Guo P., Zuo H., Chen L., Liu X., Zhao P., Xia Q. (2017). Preparation and characterization of silk sericin/PVA blend film with silver nanoparticles for potential antimicrobial application. Int. J. Biol. Macromol..

[13] Doyan A., Susilawati S., Prayogi S., Bilad M.R., Arif M.F., Ismail N.M. (2021). Polymer Film Blend of Polyvinyl Alcohol, Trichloroethylene and Cresol Red for Gamma Radiation Dosimetry. Polymers.

[14] Akhlaq M., Azad A.K., Ullah I., Nawaz A., Safdar M., Bhattacharya T., Uddin A.B.M.H., Abbas S.A., Mathews A., Kundu S.K. (2021). Methotrexate-Loaded Gelatin and Polyvinyl Alcohol (Gel/PVA) Hydrogel as a pH-Sensitive Matrix. Polymers.

[15] Khan N.A., Niazi M.B.K., Sher F., Jahan Z., Noor T., Azhar O., Rashid T., Iqbal N. (2021). Metal Organic Frameworks Derived Sustainable Polyvinyl Alcohol/Starch Nanocomposite Films as Robust Materials for Packaging Applications. Polymers.

[16] Dong X., Zhao S.X., Yin X.L., Wang H.Y., Wei Z.G., Zhang Y.Q. (2020). Silk sericin has significantly hypoglycaemic effect in type 2 diabetic mice via anti-oxidation and anti-inflammation. Int. J. Biol. Macromol..

[17] Lee S.J., Nah H., Heo D.N., Kim K.-H., Seok J.M., Heo M., Moon H.-J., Lee D., Lee J.S., An S.Y. (2020). Induction of osteogenic differentiation in a rat calvarial bone defect model using an In situ forming graphene oxide incorporated glycol chitosan/oxidized hyaluronic acid injectable hydrogel. Carbon.

[18] Jing X., Mi H.-Y., Napiwocki B.N., Peng X.-F., Turng L.-S. (2017). Mussel-inspired electroactive chitosan/graphene oxide composite hydrogel with rapid self-healing and recovery behavior for tissue engineering. Carbon.

[19] Shen T., Dai K., Yu Y., Wang J., Liu C. (2020). Sulfated chitosan rescues dysfunctional macrophages and accelerates wound healing in diabetic mice. Acta Biomater..

[20] Fonseca-García A., Caicedo C., Jiménez-Regalado E.J., Morales G., Aguirre-Loredo R.Y. (2021). Effects of Poloxamer Content and Storage Time of Biodegradable Starch-Chitosan Films on Its Thermal, Structural, Mechanical, and Morphological Properties. Polymers.

[21] Sánchez-Cardona Y., Echeverri-Cuartas C., López M., Moreno-Castellanos N. (2021). Chitosan/Gelatin/PVA Scaffolds for Beta Pancreatic Cell Culture. Polymers.

[22] Thamer B.M., Esmail G.A., Al-Dhabi N.A., Moydeen A., Arasu M.V., Al-Enizi A.M., El-Newehy M.H. (2021). Fabrication of biohybrid electrospun nanofibers for the eradication of wound infection and drug-resistant pathogens. Colloids Surf. A Physicochem. Eng. Asp..

[23] Cobos M., De-La-Pinta I., Quindós G., Fernández M.J., Fernández M.D. (2019). One-step eco-friendly synthesized silver-graphene oxide/poly(vinyl alcohol) antibacterial nanocomposites. Carbon.

[24] Zhou L., Chen F., Hou Z., Chen Y., Luo X. (2021). Injectable self-healing CuS nanoparticle complex hydrogels with antibacterial, anti-cancer, and wound healing properties. Chem. Eng. J..

[25] Khan M.I., Paul P., Behera S.K., Jena B., Tripathy S.K., Stålsby Lundborg C., Mishra A. (2020). To decipher the antibacterial mechanism and promotion of wound healing activity by hydrogels embedded with biogenic Ag@ZnO core-shell nanocomposites. Chem. Eng. J..

[26] Bhardwaj N., Bhardwaj S.K., Mehta J., Kim K.H., Deep A. (2017). MOF-Bacteriophage Biosensor for Highly Sensitive and Specific Detection of Staphylococcus aureus. ACS Appl. Mater. Interfaces.

[27] Sun T., Hao S., Fan R., Qin M., Chen W., Wang P., Yang Y. (2020). Hydrophobicity-Adjustable MOF Constructs Superhydrophobic MOF-rGO Aerogel for Efficient Oil-Water Separation. ACS Appl. Mater. Interfaces.

[28] Zhao H., Xia Q., Xing H., Chen D., Wang H. (2017). Construction of Pillared-Layer MOF as Efficient Visible-Light Photocatalysts for Aqueous Cr(VI) Reduction and Dye Degradation. ACS Sustain. Chem. Eng..

[29] Abánades Lázaro I., Forgan R.S. (2019). Application of zirconium MOFs in drug delivery and biomedicine. Coord. Chem. Rev..

[30] Wang Y., Ying T., Li J., Xu Y., Wang R., Ke Q., Shen S.G.F., Xu H., Lin K. (2020). Hierarchical micro/nanofibrous scaffolds incorporated with curcumin and zinc ion eutectic metal organic frameworks for enhanced diabetic wound healing via anti-oxidant and anti-inflammatory activities. Chem. Eng. J..

[31] Guan Q.L., Han C., Bai F.Y., Liu J., Xing Y.H., Shi Z., Sun L.X. (2020). Bismuth-MOF based on tetraphenylethylene derivative as a luminescent sensor with turn-off/on for application of Fe^3+^ detection in serum and bioimaging, as well as emissive spectra analysis by TRES. Sens. Actuators B Chem..

[32] Li X., Li X., Li D., Zhao M., Wu H., Shen B., Liu P., Ding S. (2020). Electrochemical biosensor for ultrasensitive exosomal miRNA analysis by cascade primer exchange reaction and MOF@Pt@MOF nanozyme. Biosens. Bioelectron..

[33] Han D., Li Y., Liu X., Yeung K.W.K., Zheng Y., Cui Z., Liang Y., Li Z., Zhu S., Wang X. (2021). Photothermy-strengthened photocatalytic activity of polydopamine-modified metal-organic frameworks for rapid therapy of bacteria-infected wounds. J. Mater. Sci. Technol..

[34] Lu X., Ye J., Zhang D., Xie R., Bogale R.F., Sun Y., Zhao L., Zhao Q., Ning G. (2014). Silver carboxylate metal-organic frameworks with highly antibacterial activity and biocompatibility. J. Inorg. Biochem..

[35] Lu Z., Gao J., He Q., Wu J., Liang D., Yang H., Chen R. (2017). Enhanced antibacterial and wound healing activities of microporous chitosan-Ag/ZnO composite dressing. Carbohydr. Polym..

[36] Travlou N.A., Algarra M., Alcoholado C., Cifuentes-Rueda M., Labella A.M., Lázaro-Martínez J.M., Rodríguez-Castellón E., Bandosz T.J. (2018). Carbon Quantum Dot Surface-Chemistry-Dependent Ag Release Governs the High Antibacterial Activity of Ag-Metal–Organic Framework Composites. ACS Appl. Bio Mater..

[37] Zhang M., Wang G., Wang D., Zheng Y., Li Y., Meng W., Zhang X., Du F., Lee S. (2021). Ag@MOF-loaded chitosan nanoparticle and polyvinyl alcohol/sodium alginate/chitosan bilayer dressing for wound healing applications. Int. J. Biol. Macromol..

[38] Berchel M., Gall T.L., Denis C., Hir S.L., Quentel F., Elléouet C., Montier T., Rueff J.-M., Salaün J.-Y., Haelters J.-P. (2011). A silver-based metal–organic framework material as a ‘reservoir’ of bactericidal metal ions. New J. Chem..

[39] Venkataprasanna K.S., Prakash J., Vignesh S., Bharath G., Venkatesan M., Banat F., Sahabudeen S., Ramachandran S., Devanand Venkatasubbu G. (2020). Fabrication of Chitosan/PVA/GO/CuO patch for potential wound healing application. Int. J. Biol. Macromol..

[40] Zhao R., Kong W., Sun M., Yang Y., Liu W., Lv M., Song S., Wang L., Song H., Hao R. (2018). Highly Stable Graphene-Based Nanocomposite (GO-PEI-Ag) with Broad-Spectrum, Long-Term Antimicrobial Activity and Antibiofilm Effects. ACS Appl. Mater. Interfaces.

[41] Yadav A., Kumar R., Pandey U.P., Sahoo B. (2021). Role of oxygen functionalities of GO in corrosion protection of metallic Fe. Carbon.

[42] Lee S.-Y., Moore R.B., Mahajan R.L. (2021). An Al-assisted GO/rGO Janus film: Fabrication and hygroscopic properties. Carbon.

[43] Khalil W.F., El-Sayyad G.S., El Rouby W.M.A., Sadek M.A., Farghali A.A., El-Batal A.I. (2020). Graphene oxide-based nanocomposites (GO-chitosan and GO-EDTA) for outstanding antimicrobial potential against some Candida species and pathogenic bacteria. Int. J. Biol. Macromol..

[44] Wang J.C., Karmakar R.S., Lu Y.J., Chan S.H., Wu M.C., Lin K.J., Chen C.K., Wei K.C., Hsu Y.H. (2019). Miniaturized Flexible Piezoresistive Pressure Sensors: Poly(3,4-ethylenedioxythiophene):Poly(styrenesulfonate) Copolymers Blended with Graphene Oxide for Biomedical Applications. ACS Appl. Mater. Interfaces.

[45] Kwon Y., Liu M., Castilho C., Saleeba Z., Hurt R., Külaots I. (2021). Controlling pore structure and conductivity in graphene nanosheet films through partial thermal exfoliation. Carbon.

[46] Shuai Y., Yang S., Li C., Zhu L., Mao C., Yang M. (2017). In situ protein-templated porous protein-hydroxylapatite nanocomposite microspheres for pH-dependent sustained anticancer drug release. J. Mater. Chem. B.

[47] Usman A., Hussain Z., Riaz A., Khan A.N. (2016). Enhanced mechanical, thermal and antimicrobial properties of poly(vinyl alcohol)/graphene oxide/starch/silver nanocomposites films. Carbohydr. Polym..

[48] Rui L., Xie M., Hu B., Zhou L., Yin D., Zeng X. (2017). A comparative study on chitosan/gelatin composite films with conjugated or incorporated gallic acid. Carbohydr. Polym..

[49] Ding L., Shan X., Zhao X., Zha H., Chen X., Wang J., Cai C., Wang X., Li G., Hao J. (2017). Spongy bilayer dressing composed of chitosan-Ag nanoparticles and chitosan-Bletilla striata polysaccharide for wound healing applications. Carbohydr. Polym..

[50] Sun J., Jiang H., Wu H., Tong C., Pang J., Wu C. (2020). Multifunctional bionanocomposite films based on konjac glucomannan/chitosan with nano-ZnO and mulberry anthocyanin extract for active food packaging. Food Hydrocoll..

[51] Omidi S., Kakanejadifard A. (2019). Modification of chitosan and chitosan nanoparticle by long chain pyridinium compounds: Synthesis, characterization, antibacterial, and antioxidant activities. Carbohydr. Polym..

[52] Sasidharan A., Panchakarla L.S., Sadanandan A.R., Ashokan A., Chandran P., Girish C.M., Menon D., Nair S.V., Rao C.N., Koyakutty M. (2012). Hemocompatibility and macrophage response of pristine and functionalized graphene. Small.

[53] Yuan H., Chen L., Hong F.F. (2020). A Biodegradable Antibacterial Nanocomposite Based on Oxidized Bacterial Nanocellulose for Rapid Hemostasis and Wound Healing. ACS Appl. Mater. Interfaces.

[54] Liang Y., Zhao X., Hu T., Chen B., Yin Z., Ma P.X., Guo B. (2019). Adhesive Hemostatic Conducting Injectable Composite Hydrogels with Sustained Drug Release and Photothermal Antibacterial Activity to Promote Full-Thickness Skin Regeneration During Wound Healing. Small.

[55] Guan L.Z., Patino J., Cuadrado-Collados C., Tamayo A., Gutierrez M.C., Ferrer M.L., Silvestre-Albero J., Del Monte F. (2019). Carbon-GO Composites with Preferential Water versus Ethanol Uptake. ACS Appl. Mater. Interfaces.

[56] Ju S., Zhang F., Duan J., Jiang J. (2020). Characterization of bacterial cellulose composite films incorporated with bulk chitosan and chitosan nanoparticles: A comparative study. Carbohydr. Polym..

[57] Wang X., Tang J., Huang J., Hui M. (2020). Production and characterization of bacterial cellulose membranes with hyaluronic acid and silk sericin. Colloids Surf. B Biointerfaces.

[58] Miguel S.P., Moreira A.F., Correia I.J. (2019). Chitosan based-asymmetric membranes for wound healing: A review. Int. J. Biol. Macromol..

[59] Chen Z., Yao X., Liu L., Guan J., Liu M., Li Z., Yang J., Huang S., Wu J., Tian F. (2017). Blood coagulation evaluation of N-alkylated chitosan. Carbohydr. Polym..

[60] Sridhar R., Sundarrajan S., Vanangamudi A., Singh G., Matsuura T., Ramakrishna S. (2014). Green Processing Mediated Novel Polyelectrolyte Nanofibers and Their Antimicrobial Evaluation. Macromol. Mater. Eng..

[61] Chandrasekaran A.R., Venugopal J., Sundarrajan S., Ramakrishna S. (2011). Fabrication of a nanofibrous scaffold with improved bioactivity for culture of human dermal fibroblasts for skin regeneration. Biomed. Mater..

